# Advances in Ultrahigh Throughput Hit Discovery with Tandem Mass Spectrometry Encoded Libraries

**DOI:** 10.1021/jacs.3c04899

**Published:** 2023-08-09

**Authors:** J. Miguel Mata, Edith van der Nol, Sebastian J. Pomplun

**Affiliations:** † Leiden University, 2333 CC Leiden, The Netherlands

## Abstract

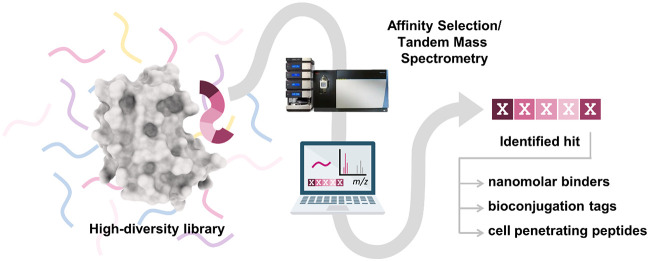

Discovering new bioactive molecules is crucial for drug development. Finding a hit compound for a new drug target usually requires screening of millions of molecules. Affinity selection based technologies have revolutionized early hit discovery by enabling the rapid screening of libraries with millions or billions of compounds in short timeframes. In this Perspective, we describe recent technology breakthroughs that enable the screening of ultralarge synthetic peptidomimetic libraries with a barcode-free tandem mass spectrometry decoding strategy. A combination of combinatorial synthesis, affinity selection, automated *de novo* peptide sequencing algorithms, and advances in mass spectrometry instrumentation now enables hit discovery from synthetic libraries with over 100 million members. We provide a perspective on this powerful technology and showcase success stories featuring the discovery of high affinity binders for a number of drug targets including proteins, nucleic acids, and specific cell types. Further, we show the usage of the technology to discover synthetic peptidomimetics with specific functions and reactivity. We predict that affinity selection coupled with tandem mass spectrometry and automated *de novo* decoding will rapidly evolve further and become a broadly used drug discovery technology.

## Introduction

Discovering a potent binder for a novel drug target often requires sifting through millions of molecules. Affinity selection (AS) is a powerful technology that allows for the swift identification of high-affinity ligands to a given target. Typically, an AS experiment involves mixing a compound library containing millions or even billions of compounds with a target molecule (e.g., a pharmacologically relevant protein) and through a physical separation step, such as protein immobilization,^1−3^ filtration,^4^ or size exclusion chromatography (SEC),^5−8^ binders from the library are separated from nonbinders. The libraries can consist of ribosomally produced peptides,^9−12^ combinatorically prepared synthetic molecules,^13−16^ or mixtures of natural compounds.^17^ Compared to classical high throughput screenings (HTS), the gold standard in the pharmaceutical industry, AS provides a much faster way to explore a vast chemical space and identify new ligands for drug targets.

**Figure 1:**
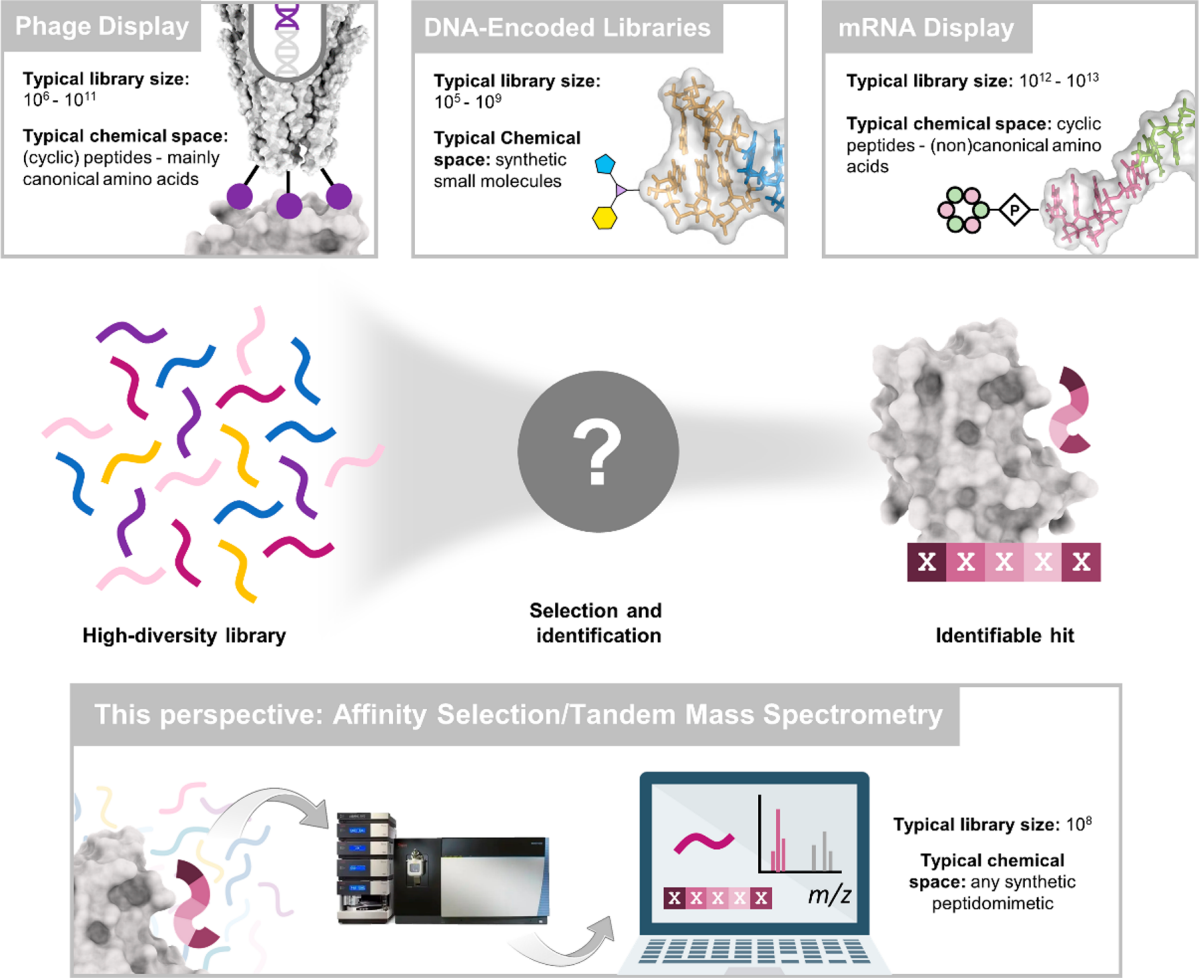
Overview of different affinity selection technologies for hit discovery. In affinity selection (AS) technologies, millions or billions of compounds can be mixed with a given target to select high affinity binders. Top: established AS platforms using genetic barcodes for hit identification. From left to right: phage display, DNA-encoded libraries and mRNA-display. Bottom: affinity selection coupled to tandem mass spectrometry. This technology does not require compound barcoding. All library members are “self-encoded” by their tandem MS/MS spectra. High sensitivity instrumentation and automated *de novo* sequencing software enable the screening of over 100 million molecules per experiment with this technology.

A critical step in affinity selection workflows is the accurate identification of binders, selected from a vast library. A widely adopted approach involves barcoding each library member with genetic material, followed by decoding the barcoded compound using DNA sequencing (Figure 1).^18^

In the Nobel Prize awarded phage-display technology, peptides (or proteins) are displayed on the envelope of bacteriophages and panned against immobilized target biomolecules (Figure 1).^9,10,19,20^ After one (or multiple) round(s) of selection, the phage genome is amplified and sequenced to decode the identity of the displayed peptide. Peptide libraries generated by this method are mainly limited to canonical amino acids, but cyclization strategies have produced interesting cyclic and bicyclic peptide scaffolds.^3,21,22^ mRNA-display directly links macrocyclic peptides to their messenger RNA (mRNA) (Figure 1).^11^ Several technology breakthroughs have enabled the incorporation of a wide range of noncanonical amino acids into mRNA-display libraries, especially in the random nonstandard peptides integrated discovery (RaPID) system, developed in the Suga Lab.^11^ Peptides and peptidomimetic compounds are particularly interesting for the targeting of disease related protein–protein interactions (PPIs).^23^

In the DNA-encoded library technology, synthetic molecules are linked to DNA strands (Figure 1).^24,25^ Different synthesis steps are paired to the ligation of DNA fragments that encode for specific building blocks or transformations. Compared with display technologies, DNA-encoded libraries (DELs) are not limited to peptide-based compounds and building blocks suitable for ribosomal incorporation. Phage-display, mRNA-display, and DELs have definitely revolutionized the speed of drug hit finding and are widely used in academic laboratories, biotech companies, and the pharmaceutical industry.

Affinity selection–mass spectrometry (AS–MS) is a technology that enables selections without requiring compound barcoding with genetic material.^26−30^ After the AS step, the enriched mixture of potential binders is analyzed by liquid chromatography–mass spectrometry (LC–MS) to retrieve the hit compounds’ identity. The advantages of these “self-encoded libraries” (SELs) relate to the absence of barcoding tags that can interfere with the affinity selection process. Chemically very diverse libraries can be used as there are no limitations for ribosomal production or DNA-compatible chemistry.^31−35^ Two well-established AS–MS technologies are SpeedScreen^36,37^ and the Automated Ligand Identification System (ALIS)^6^ respectively developed by Novartis Pharma and NeoGenesis Pharmaceutical. The ALIS system has been successfully used to identify ligands for multiple protein targets^38−41^ and even for several nucleic acid targets.^32,33,42,43^ AS–MS workflows have been utilized also in the context of dynamic combinatorial libraries to discover high affinity lectins.^44^ Intrinsic limitations of AS–MS are related to the hit readout method. Structurally diverse molecules with the same exact mass are difficult to be distinguished, therefore limiting the readout capacity to several hundred to a few thousand compounds per sample pool.^31,36,45,46^ Automated procedures and infrastructures, however, can accelerate the process and screen many samples per day, reaching library sizes between 10^5^ and 10^6^ members.^41,47^ With this combination of affinity selection and automation, AS–MS can be seen as an intermediate strategy between classical HTS and massive library selections. For a more detailed view on the AS–MS field, we refer to a recent review article by Prudent et al.^45^

While decoding complex mixtures of nonbarcoded compounds represents a significant challenge, the preparation of ultralarge combinatorial libraries with millions of synthetic compounds (e.g., peptidomimetics) is relatively straightforward and has been possible since the early 90s.^15^ Early synthetic combinatorial libraries had to be screened in arrayed formats^48^ or using the “one-bead one-compound” (OBOC) approach.^15^ Due to the difficulties in decoding potential hit compounds, OBOC based screenings suffered from cumbersome workflows, diminishing the interest in the technology over the years.

The decoding of synthetic peptide libraries requires specialized *de novo* sequencing software. The database search approach, commonly used for peptide mixtures in proteomic experiments,^49,50^ is not feasible, given that the library peptides cannot be related to any known genome or protein sequences. To decode “unknown” peptide sequences a number of automated *de novo* sequencing programs have been developed.^51−53^ These software packages enable the automated sequencing and enumeration of sequences contained in a complex mixture of peptides analyzed in an LC–MS/MS run.

In this Perspective, we describe how a combination of combinatorial synthesis, affinity selection, automated *de novo* peptide sequencing algorithms, and advances in mass spectrometry instrumentation has enabled a breakthrough for the capabilities of hit discovery from barcode-free, self-encoded synthetic libraries (SELs) with up to 10^8^–10^9^ members (Figure 1).^1,54^ We explain the key technology features of the workflow and give an overview of success stories using affinity selection coupled with tandem mass spectrometry (AS–MS/MS) to identify high affinity ligands for several disease related proteins, nucleic acids, and specific cell types. Beyond the discovery of binders, the technology has been used to identify functional peptide sequences with, e.g., cell-penetrating properties or sequence specific reactivity. Very recently, synthetic peptides were used as barcodes for small molecule libraries, enabling a novel type of encoded library selections for nonpeptidic compounds.

## Essential Technology Features for AS–MS/MS Discovery Workflows

The main steps for the SEL AS–MS/MS workflow comprise combinatorial library synthesis, affinity selection, and peptide sequencing. In the following paragraphs, we give a technical overview on these technologies.

### Combinatorial Library Synthesis—How to Get Hundreds of Millions of Compounds?

**Figure 2:**
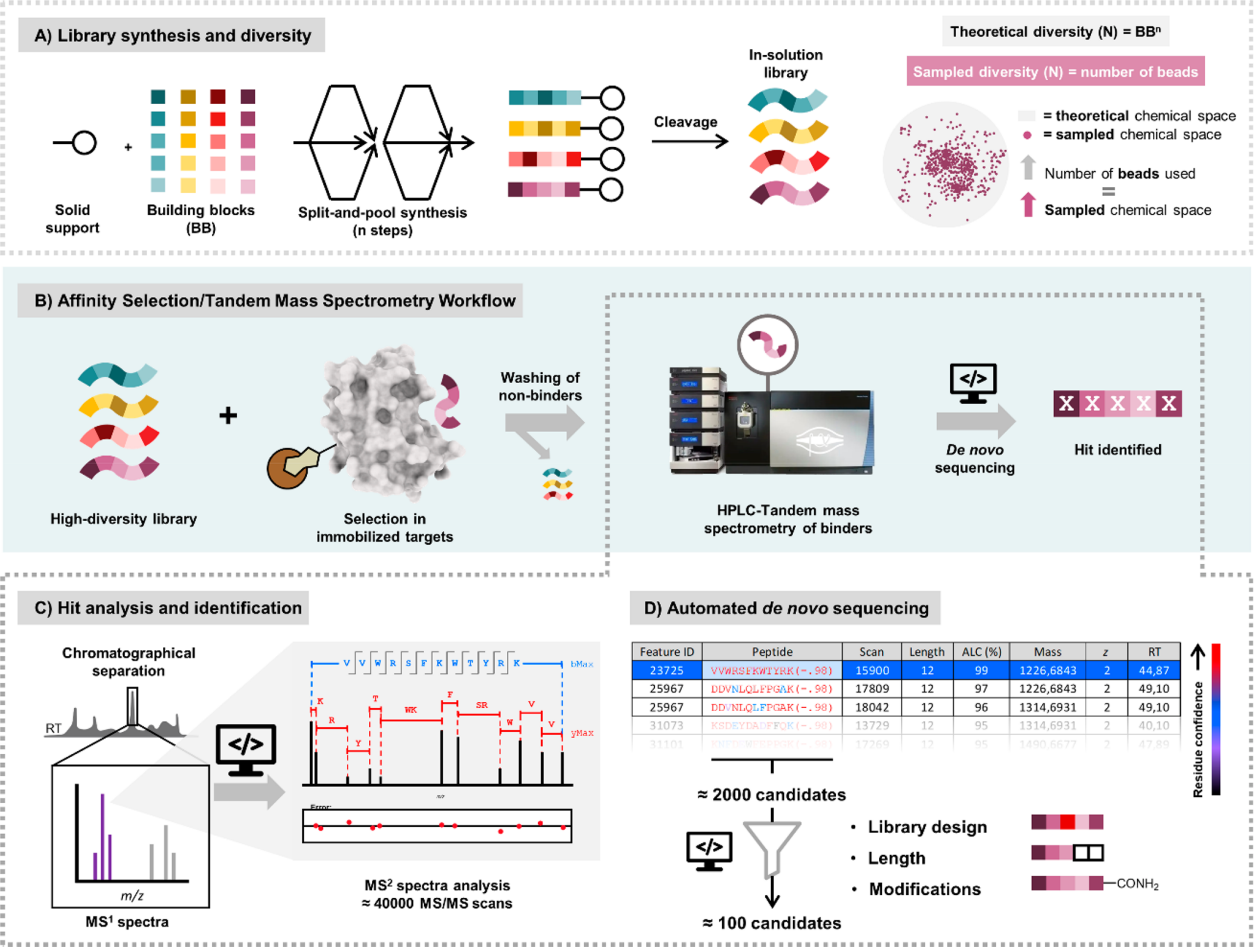
Affinity selection: tandem mass spectrometry workflow overview. (A) Combinatorial synthesis: the split and pool methodology enables the rapid preparation of large combinatorial libraries. The library diversity depends on number of building blocks, coupling steps (or peptide length) and is ultimately limited by the number of beads used. (B) A general AS–MS/MS workflow consist of mixing a library with a target, separating binders from nonbinders, analyzing the bound fraction by LC–MS/MS and utilizing *de novo* sequencing software to retrieve the identity of the hit compounds. (C, D) An LC–MS/MS run, depending on run time and instrument settings results in thousands of MS and MS/MS spectra. Automated *de novo* sequencing software, such as PEAKS,^51^ analyses all spectra and delivers a list with possible sequence candidates. Additional filtering steps can help to restrict the final candidate list to sequences that fit the library design (e.g., C-term amidation, peptide length, building block selection.).^54^

The main stay technology for the preparation of combinatorial synthetic peptidomimetic libraries is solid phase “split and pool” synthesis (Figure 2a).^1,14,15^ The process begins by splitting a large number of solid phase beads into smaller portions. Each of these portions is then treated with a specific building block (e.g., amino acid). After incorporation of the first building block, the portions are combined (pooled), mixed, and then split again into smaller groups. Each of these groups is treated again with the next building block. This process is repeated several times until the desired peptide length is obtained. In the split and pool methodology, on each resin bead, a single identity peptide sequence is assembled, resulting in an OBOC library.

The theoretical diversity of a combinatorial library results from (the number of building blocks) to the power of (the residue number) (Figure 2a). Since a single peptide identity can be assembled on each bead, if the theoretical diversity is higher than the amount of beads, only a fraction of all possible sequence combinations is sampled. The number of beads used for the library preparation depends on bead size and quantity. One gram of monosized tentagel resin has 3 million beads in case of 90 μm particles and 150 million beads with 30 μm particles. A 30 μm resin bead, with a typical loading capacity of 0.25 mmol/g, contains ∼4 pmol peptide. The sensitivity of the library analysis methodology determines which beads can be used for the combinatorial library preparation. Early library hit identification methodologies required more material and thus larger beads. Preparing libraries exceeding a few million members using large beads was impractical, as it would have required excessive amounts of solid phase resin. The sensitivity of modern mass spectrometry, however, now enables the identification of peptide quantities of ∼1 fmol, allowing for the use of ultrahigh diversity libraries, prepared on smaller resin beads.^1,55,56^

For a quality control of the library synthesis a number of random beads can be picked and analyzed.^1,54^ This validation represents an important advantage over DNA-encoded libraries, in which there is no direct way to assay the quality of library synthesis. Originally, OBOC libraries have been directly screened on beads.^14^ However, most of the ultrahigh throughput workflows that we will summarize in this Perspective cleave their libraries from the beads and use them as homogeneous mixtures in solution screenings.

Synthetic libraries have an advantage over molecular biology approaches, as virtually any noncanonical building block can be incorporated, enabling the rapid synthesis of peptide pools with novel properties and iterative libraries for affinity maturation. A new library can be prepared in as few as 1 or 2 days. These features make combinatorial libraries an attractive option for the discovery of peptidomimetics with diverse chemical space, proteolytic stability, low immunogenicity, and better binding profiles for challenging targets.

### Affinity Selection—Finding the Needle in the Haystack

The affinity selection (AS) step aims to physically separate binders from nonbinders. A common strategy (also used in most phage- or mRNA-display and DNA-encoded library selections) is panning against immobilized targets (Figure 2b).^1^ The target can be on beads (e.g., magnetic or agarose) or surfaces. The immobilization chemistry often relies on streptavidin–biotin, His-tag, or covalent linkages.^57^ To avoid the selection of unspecific (“sticky”) binders, the selection buffer usually contains blocking agents such as BSA or fetal bovine serum and surfactants. More or less stringent washings are performed to remove nonbinders from the sample. The binders are usually recovered using denaturing conditions such as heat, acid, organic solvents, or chaotropic buffers (such as 6 M guanidine).^1,58,59^

Size exclusion chromatography (SEC) is another mainstay AS technology.^5^ A library and a target are mixed in-solution and subjected to SEC. Library members with a high binding affinity elute with the target fraction, separated from the much smaller nonbinding library residues. AS–MS approaches often rely on the SEC technology because it is practical to automate the process and to directly connect the SEC to an LC–MS instrument.^31^

In any AS experiment, compounds with a high affinity are separated from compounds that do not have an affinity for the target. Usually, each individual library member is present at a minute concentration (often pico- or femtomolar). Therefore, the target concentration drives the binding equilibrium. The target often is used at 100–1000 nM.^60^ When the immobilized target is utilized for the selection, the high local concentration on the surface can lead to a rebound effect and with that to more efficient capture of binders.

### Automated *De Novo* Sequencing for Unambiguous Hit Identification

Predicting how many peptide sequences are captured during the affinity selection step is impossible. The sample eluted after AS could contain a handful or hundreds of potential hits. When analyzing this sample of unknown complexity by LC–MS/MS, depending on the run length and scan speed, several thousand or tens of thousands of MS scans are performed. A manual analysis of such an amount of data is not feasible. At the same time, synthetic peptide libraries do not allow for database searches, as it is done for proteomics peptide samples.^49^ As described above, the possible combinations of peptides in a combinatorial library depend on the peptide length and number of building blocks. Even with rather short, 10-mer, peptides, and using solely canonical amino acids the theoretical combinations exceed the trillions (20^10^ = 10^13^). Many of these sequences do not exist in nature and are not related to any genomic protein sequence. The usage of noncanonical building block leads to an even more complex chemical space. While for proteomics experiments incomplete MS/MS annotations can be filled up with existing database or sequence information, a synthetic peptide requires complete annotation of its fragmentation spectra for accurate decoding.

Automated *de novo* sequencing, however, provides the possibility of extracting sequence information from MS/MS spectra without the use of database searching (Figure 2C,D). Originally, software packages including PEAKS,^51^ PepNovo,^52^ and Lutefisk^53^ were developed for the identification of proteins from unknown genomes. The underlying algorithms take into account many possible fragmentation patterns of the peptide backbones. Possible combinations of amino acid sequences fitting the precursor mass and the fragmentation spectra peaks are computed, and the most likely sequences are proposed in a ranked output.

Vinogradov et al. have demonstrated the use of the PEAKS *de novo* sequencing software for the automated decoding of mixtures of synthetic peptides.^54^ The sample resulting from an affinity selection experiment prior to analysis is of unknown complexity. From a handful of potential binders to several hundreds of unique sequences, it could be selected. In their investigation, Vinogradov et al. mimicked samples resulting from an affinity selection by preparing mixtures with a defined number of synthetic peptides, as subsets from high diversity libraries. Via a single nanoLC–MS/MS run, followed by automated PEAKS *de novo* sequencing and a refinement protocol, to remove duplicates and sequences that do obviously not fit the initial library design, the authors could decode up to 600 peptides with over 85% sequence identification rate. For a thorough comparison between the performance of database searching and *de novo* sequencing ,we refer to the recent article by Koh et al.^61^

The ability to prepare peptidomimetics with unnatural amino acids is one of the major advantages of synthetic library preparation over molecular biology-based technologies. Mass spectrometry-based decoding, if not relying on database searching, is agnostic in terms of from what building blocks the peptidomimetic sequence is built. Vinogradov et al. demonstrated that the nanoLC–MS/MS + PEAKS based decoding approach is also feasible for synthetic mixtures with a high number of unnatural building blocks. The ability to correctly identifying the sequences from complex mixtures with hundreds of synthetic peptide based compounds was further confirmed in further studies by Pomplun et al.^16,62^ These observations indicated that the approach could be utilized to decode structurally diverse samples of polypeptides and peptidomimetics resulting from affinity selection workflows.

## Success Stories of Hit Discovery with AS–MS/MS

### Discovery of High Affinity Binders for Proteins and Nucleic Acids with in Solution AS/MS–MS

**Figure 3:**
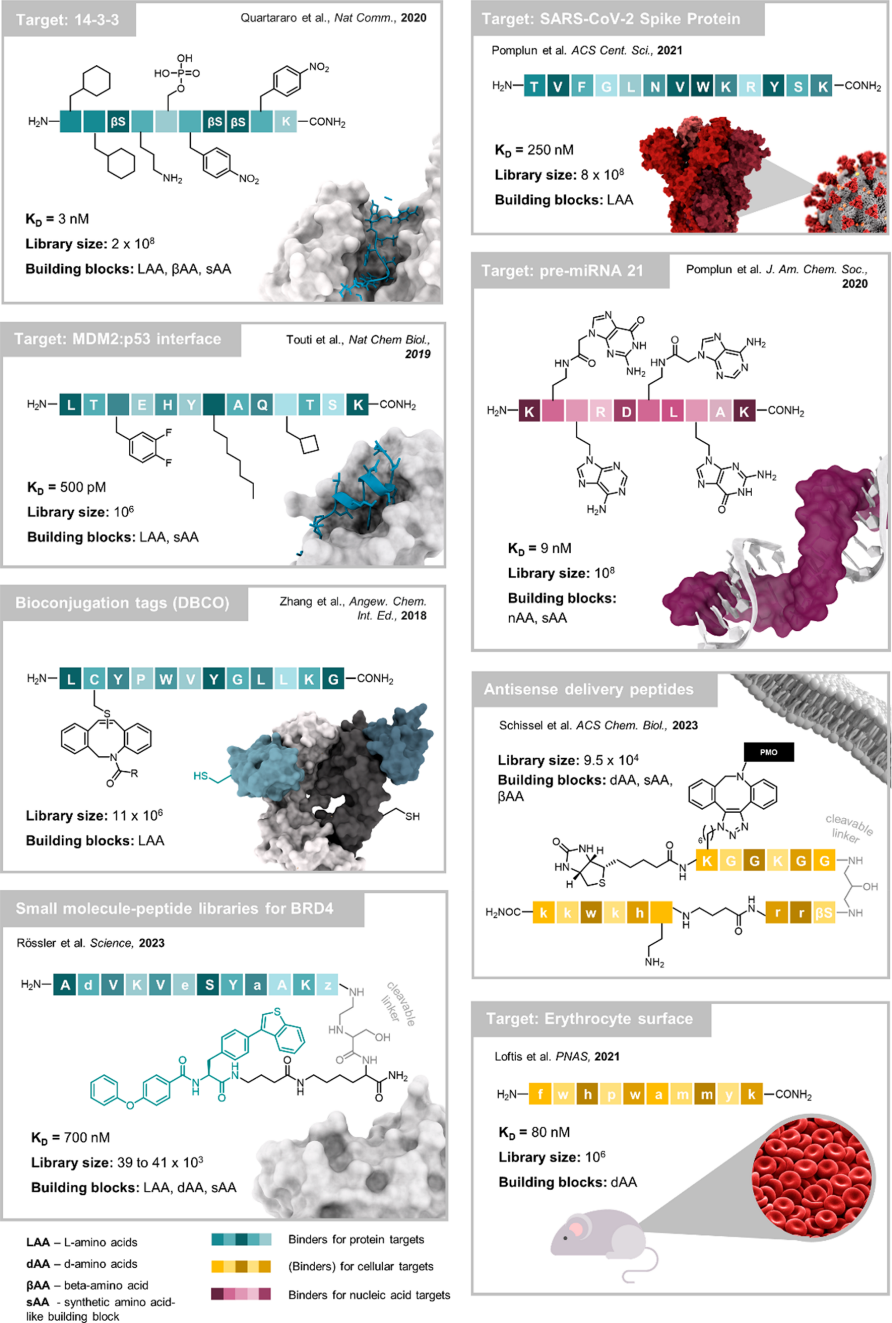
Examples of peptide and peptidomimetic hits discovered via AS–MS/MS. The technology platform has been used to discover high affinity binders for multiple pharmacologically relevant proteins^1,5,63^ and nucleic acids.^16^*In vivo* selections resulted in the identification of erythrocyte binders.^4^ Functional screenings enabled the identification of potent cell penetrating peptides^65^ and bioconjugation tags.^66^

Combining combinatorial library synthesis, affinity selection, and the recent advances in automated *de novo* sequencing enabled discovery workflows with ultralarge and chemically diverse peptidomimetic libraries (Figure 3). Quartararo et al. developed a robust workflow for affinity selections with up to 200 million synthetic peptides or peptidomimetics per sample.^1^ The possibility to use very small amounts of each library member is instrumental to reach such a large library size. The authors demonstrated that ∼1–10 fmol is sufficient to enable AS pull-down and unambiguous MS/MS based sequencing of high affinity binders. The AS was performed with targets immobilized on magnetic beads. Taking binding equilibria into consideration, the target concentration (∼100 nM) represents an important factor for the affinity range of selected binders, which, indeed, mostly have *K*_D_s < 100 nM. Using in solution AS followed by nanoLC–MS/MS + PEAKS based decoding the authors discovered nanomolar binders for the proteins 12ca5, MDM2, and 14–3–3. Quartararo et al. demonstrated the importance of screening libraries with sizes exceeding 100 million compounds for the identification of nanomolar binders. Interestingly, when selection experiments with a library size of 10^9^ were performed, fewer binders were identified compared to the experiments with 10^8^ compounds. The authors excluded competition for target protein and MS sensitivity as causes for this phenomenon. The actual reason for this limitation remains unknown. The workflow was performed with both canonical and noncanonical peptide libraries including D- and β-amino acids and several synthetic side chains. In the selection for 14–3–3 binders, a phosposerine and β-amino acids were crucial for binding, as confirmed by a 3D cocrystal structure (PDB_6TCH, Figure 3). Overall, this work demonstrates the feasibility of AS–MS/MS with library sizes approximating those of phage-display. Working with library sizes exceeding 10^8^ compounds remains a challenge to be solved for AS–MS/MS.

AS–MS/MS enables the rapid identification of first class binders for novel targets. In the early stages of the global COVID-19 pandemic, Pomplun et al. used AS–MS/MS to identify peptides targeting the SARS-CoV-2-Spike-Protein (Figure 3). Three peptide sequences with dissociation constants in the nanomolar range were identified for SARS-CoV-2 after screening 800 million peptides (in pools of 200 millions) all sharing a motif at the *N*-terminus.^63^ The peptides were not able to inhibit the virus from binding to human cells because they do not bind to the ACE-2 binding site on the SARS-CoV-2-Spike-Protein. Isolating peptides with affinity for a “nonfunctional” binding site of the target is an intrinsic limitation to affinity selection workflows, which do only select for binding affinity and not for activity. However, high affinity tools with good selectivity, such as these peptides, were proposed as the starting point for the development of practical diagnostic tests. A subsequent mRNA-display selection, performed by the group of Payne with 10^12^ cyclic peptides, identified several binders for the SARS-CoV-2-Spike-Protein.^64^ The only hit, validated for affinity and selectivity, was a parent peptide of the AS–MS/MS screen with a motif comprising five shared residues. This “consensus discovery” of two independent groups utilizing different technologies highlights the AS–MS/MS workflow as a powerful discovery method, on par with display-based workflows.

In a selection for binders to the angiotensin converting enzyme (ACE2), both canonical and noncanonical libraries each containing 200 million peptides were utilized.^67^ A comparable number of binders with equal nanomolar affinity ranges was found, but the most potent noncanonical peptide binder showed enhanced serum stability compared to the highest affinity canonical binder.

In 2020, Pomplun et al. further expanded the chemical diversity of peptidomimetic libraries by incorporating nucleobase side chains to target pre-microRNA hairpins.^16^ The authors synthesized 12 amino acid building blocks connected to nucleobase side chains via different linkers. A test experiment with 768 nucleobase peptides demonstrated that MS/MS could be used to efficiently decode biohybrids with a high density of nucleobase modifications. Surprisingly, variants in which the nucleobases were connected via isopeptide bonds to the backbone could also be sequenced, utilizing a combination of collision induced dissociation (CID) and electron triggered dissociation (ETD). The AS–MS/MS screening of a library with 100 million biohybrids led to the identification of binders with a single digit dissociation constant in the nanomolar range for two oncogenic noncoding RNAs (pre-miRNA21 and pre-miRNA-155, respectively, Figure 3). The fact that only a single peptide was pulled down and identified by MS/MS sequencing remains surprising. It is likely that more binders were present in the library but were lost in the workflow or not correctly detected or sequenced in the MS/MS and data analysis steps. Further optimization of several steps of the technology might be needed. Taken together, these results showcase the use of AS–MS/MS for the high throughput discovery of sequence defined polymers with designer properties, tuned toward specific target classes.

AS–MS/MS enables rapid affinity maturation of known peptide binders, exploiting a vast chemical space of noncanonical building blocks. Touti et al. showed that AS–MS can be used to improve the binding affinity of known binders to MDM2. To separate binders from nonbinders the authors utilized size exclusion chromatography.^55^ In the first iteration starting from a one million member library where six hotspot residues were varied, 18 putative binders were found in a nanomolar range. Utilizing a competitive affinity selection procedure with a noncanonical 7000-membered library, the authors identified improved binders. As an alternative approach with focused libraries, Ye et al. performed a combinatorial alanine scanning from a known binder for MDM2.^68^ In such a library each position is either the wild type residue or an alanine, exploring not only binding hot spots but also the possibility of mutating multiple resides at a time, without losing binding affinity. This combinatorial alanine scanning approach can, e.g., identify (i, i+4) pairs that can be utilized for peptide stapling. Zhang et al. performed iterative screenings of focused libraries with a few thousand members, derived from a known binder to the leukemia-associated protein menin.^69^ By using a large set of noncanonical amino acids, a novel peptide binder with significantly reduced size but retained affinity for menin was identified. The Winssinger group reported an intriguing strategy to select high affinity peptides with a complex “suprastaple” architecture. These suprastaples are short backbone PNA moieties that via base pairing constrain an alpha helical conformation of the peptides. The authors produced a focused suprastaple-peptide library via combinatorial chemical ligation and utilized AS–MS to select variants with a high affinity for the oncoprotein MDM2.^70^

### Discovery of Cell Penetrating and Cell Selective Hits

A significant development of the AS–MS/MS technology is the transition to in cell and even *in vivo* selections. Schissel et al. developed a method for in-cell penetration selection mass spectrometry (in-cell PS–MS) that opens the possibility for delivery of therapeutically relevant cargo.^65^ Cell-penetration peptides (CPPs) are able to penetrate the cellular membrane and deliver therapeutic agents such as nucleic acids and proteins, which could not enter the cells otherwise. Schissel et al. designed a library including non-α backbones to promote endosomal escape, hydrophobic and aromatic residues for increased membrane penetration and charged residues for enhanced membrane penetration. Each library member was connected to a phosphorodiamidate morpholino oligomer (PMO) and a biotin, via an oxidatively cleavable linker (isoseramox).^62^ Cells were incubated in the library. Upon lysis, the cytosolic fraction was collected, and peptides that had been able to enter the cell were captured with streptavidin beads. The hit sequences were release via isoseramox cleavage and analyzed via LC–MS/MS and *de novo* sequencing, identifying variants with high delivery efficiency (Figure 3).

An *in vivo* application of AS–MS/MS was investigated by Loftis et al.; with the aim of identifying erythrocyte binding ligands the authors injected living mice with a D-peptide library containing one million members.^4^ A D-peptide library was chosen because of its resistance to proteolytic digestion. After the blood cells were isolated, membrane-bound peptides were analyzed by nLC–MS/MS. The C-terminal amide on library peptides unambiguously differentiated them from biological peptide sequences present in the cell matrix (Figure 3). The analysis resulted in the identification of 128 erythrocyte binders. Peptides with the highest affinity could be used to direct therapeutic proteins to erythrocytes, enabling prolonged circulation times and reduced immunogenicity. The technique might be also used to find synthetic binders for delivery to specific tissues, organs, or other cell populations.

### Discovery of Small Molecules via Peptide Encoded Libraries (PELs)

Roessler et al. leveraged the high sensitivity *de novo* peptide sequencing capabilities to develop a new type of encoded small molecule discovery platform.^71^ The authors assembled small druglike molecules on a solid support, and, connected via a cleavable linker (seramox),^62^ they built up a peptide tag encoding the identity of the small molecule. After affinity selections against disease related proteins, the linker is cleaved to release the peptide tag for LC–MS/MS analysis. The peptide tags were optimized for confident decoding by tuning the polarity and charge states. In their study, Roessler et al. show the improved chemical stability of peptide tags compared to DNA tags. Two libraries of 39 000and 41 000 members were synthesized via peptide couplings and Buchwald-Hartwig and Suzuki cross coupling reactions. Binders for three targets (Carbonic anhydrase IX, BRD4, and MDM2) were identified (Figure 3). Compared with typical DELs, the PELs reported here are rather small. For more challenging targets, it will be important to access larger PELs and optimize the technology. As a matter of fact, for larger libraries, it will be important to exclude the possibility that the high diversity peptide tag library starts to interfere with the affinity selection.

### On-Bead Selection with Ultralarge MS/MS Encoded Libraries

In the following paragraphs, we describe recent advanced applications of on-bead library screenings. As opposed to the “in-solution” library approach described above, one-bead-one-compound (OBOC) libraries can be screened with all compounds still bound to their synthesis resin particles. Target biomolecules are usually fluorescently labeled, enabling the identification of library beads bound to the target. Originally, upon incubation with the target, OBOC libraries were inspected under a microscope, and beads displaying fluorescence were picked out individually. The compounds on the hit beads were then cleaved and analyzed separately via Edman degradation or later tandem mass spectrometry. Prior to the evolution of automated *de novo* sequencing this method was necessary to enable the unbiased identification of OBOC hits. The cumbersome workflow, combined with a high number of false positives (likely due to multivalency effects on the bead surface^72^), decreased the interest for the OBOC technology in the last years. Recent technology advances, however, have enormously accelerated on-bead hit findings workflows.

Gates et al. combined automated *de novo* sequencing technology with fluorescent activated bead sorting, enabling affinity selections with millions of synthetic mirror image proteins displayed on beads.^55^ The authors used the OBOC methodology to prepare libraries with 10^8^ on-bead folded knottin proteins with a variable stretch of nine amino acid residues. The library was solely built of D-amino acids, with variants with protease resistance and reduced immunogenicity. The authors called these compounds xenoproteins. To prevent excessive multivalency effects on the bead surface, a core–shell synthesis strategy was adopted,^73^ with low density surface functionalization, and high density functionalization inside the beads, to ensure sufficient material for compound identification by mass spectrometry. To obviate the limitations of precise *de novo* MS/MS sequencing for peptides exceeding ∼15 residues, inside the beads only the variable 9-mer sequence was synthesized. In a first selection step, the library was enriched for binders by panning against immobilized target protein. The enriched fraction was then incubated with fluorescently labeled target. To separate fluorescent beads from the others, a fluorescence activated cell sorting (FACS) machine was readapted to fluorescence activated bead sorting (FABS).^74^ All hits could be cleaved as a mixture, subjected to nanoLC–MS/MS, and analyzed via automated *de novo* sequencing with PEAKS. A xenoprotein with 50 nM affinity for the hemagglutinin antibody 12ca5 was identified.

OBOC selection combined with FABS can be used to identify peptide sequences with unique reactivity. Aiming to identify a cysteine containing peptide sequence, with enhanced reactivity for bioconjugation reactions with dibenzocyclooctyne (DBCO), Zhang et al. utilized an OBOC–FABS–*de novo* sequencing workflow.^66^ They prepared a combinatorial library containing the variable motif XCXXXXX. The on-bead library was incubated with DBCO-(PEG)_4_-biotin and the beads containing reacted species were isolated by FABS after staining with a streptavidin-conjugated fluorophore. LC–MS/MS followed by *de novo* sequencing resulted in 40 putative hits. A confirmed hit sequence with validated reactivity was then further optimized for the DBCO-tag LCYPWVY- (Figure 3). Compared to a cysteine in a random sequence, the DBCO-tag has a 220-fold increased reactivity. A similar approach was leveraged to identify sequences with unique cysteine reactivity toward perfluoro aryl moieties.^75,76^ Peptide tags with chemoselective reactivity are crucial for bioconjugation approaches such as antibody drug conjugations. Combinatorial libraries combined with the described efficient selection workflows are a valuable source of information for the identification of such sequences.

Avital-Shmilovici et al. developed an alternative bead-based selection platform for synthetic peptidomimetic libraries with up to one billion members.^56^ They used used fiber-optic array scanning technology (FAST), a technology initially developed for cancer diagnostics,^77^ as a bead sorting methodology. The technology can screen up to 5 million beads per minute. The authors created a novel self-readable sequencing approach for synthetic sequence defined polymer libraries. Library monomer blocks consist each of three sub-building-blocks (e.g., canonical and noncanonical amino acids) are called “ptychs”. Pytchs are connected to each other via a phenylacetamido-methylene (PAM) linker. After the FAST selection, the PAM linkers are cleaved under basic conditions, disassembling hits in their individual ptych blocks. This chemical cleavage is used instead of MS^2^ fragmentation. Each ptych has a unique mass, allowing for unambiguous identification by high resolution mass spectrometry. The FAST ptych strategy identified high affinity binders for multiple disease relevant targets (K-Ras, ASGPR, IL6, IL6R, and TNFα).

A requirement for MS/MS sequencing is the use of linear peptides. However, cyclic and bicyclic peptides have several advantages over their linear counterparts. Cyclic peptides usually have higher proteolytic stability, and their structural preorganization makes them more suitable for the high affinity binding of challenging targets with shallow binding sites. In order to screen cyclic peptide libraries with an MS/MS based decoding workflow, a ring opening step prior to mass spectral analysis is required. Several linearization strategies have been described in the literature, including oxidative diol cleavage,^5^ photocleavage of tetrazine,^78^ oxazolidinone cleavage,^79^ and disulfide opening.^80^ Most of these studies described the linearization strategy without performing affinity selection discovery experiments.

In a recent study, Li et al. prepared a protein mimetic peptide library with rigid, bicyclic scaffolds, employing a dual linearization strategy.^81^ The bicyclic compounds were obtained by the CuAAC reaction and Ruthenium catalyzed metathesis between two allyl functionalized glutamic acid residues. They enabled linearization and subsequent MS/MS sequencing via a dual cleavage strategy: Edman degradation on the peptide’s N-terminus opened the ring formed by CuAAC and Pd-based deallylation opened the ring formed by the metathesis reaction. The library was screened against the oncogenic transcription factor MYC and a micromolar binder was identified.

## Discussion and Future Perspective

Affinity selection technologies have revolutionized early drug discovery by enabling the screening of enormous chemical space in significantly shorter timeframes and with limited need for complex facilities, compared to classical HTS. Efficient library preparation and high sensitivity decoding technology are important factors for AS with multimillion membered libraries. Genetically encoded technologies, including phage and mRNA-display and DNA encoded libraries, have been widely used and evolved over the past decades. In this Perspective, AS–MS/MS is presented as a novel technology enabling ultrahigh throughput hit discovery. Combining combinatorial solid phase synthesis with high resolution and sensitivity mass spectrometry, and automated *de novo* sequencing software now allows for the screening of peptidomimetic libraries with hundreds of millions of individual compounds. We refer to the barcode-free self-encoded libraries used in AS–MS/MS as SELs.

The accessible chemical diversity of synthetic peptidomimetic SELs is virtually unlimited, potentially making up for the lower numerical diversities compared to phage- or mRNA-display. Thousands of unnatural amino acid building blocks are commercially available and can be incorporated into synthetic libraries suitable for AS–MS/MS. The synthesis of customized building blocks tuning library properties is straightforward, and even building blocks with complex side chain modification can be detected, and sequenced with the automated procedures.^16^ We have shown the importance of these unnatural building blocks for improving target recognition,^1,5,16,69^ proteolytic stability,^67^ cell penetration,^82,83^ and in vivo behavior.^4^ In the future, a synergistic pairing between display and AS–MS/MS workflows would be a powerful strategy. First pass selections with ultrahigh diversity display libraries (e.g., 10^13^) could deliver initial hits, which may be further refined and maturated via synthetic library selections.

SELs with ∼10^8^ members enable efficient *de novo* discovery of high affinity binders. We have provided examples of the identification of nanomolar binders for a broad range of targets. Most of the *de novo* discovery experiments were performed with libraries containing ∼10^8^ members.^1,16,63,67^ Libraries with 10^9^ members were tested, but did not result in increased hit rates.^1^ Recent evidence shows that also for DNA-encoded libraries the sweet spot for the best hit identification rates is achieved with library sizes between 10^6^ and 10^8^ (as described by Satz et al.^84^ and by x-chemrx^85^). Indeed, many recently reported DNA-encoded libraries did not exceed the millions.^2,86^ While the detection and sequencing of DNA barcodes definitely have superior sensitivity compared to peptide decoding, SELs can reach diversities comparable to functional DELs. In addition, the genetic barcode is often described as a potential interfering factor in the affinity selection experiment. A tag-free technology approximating the diversities of display selections is, therefore, an important addition for the field of AS-based drug discovery.

Another consideration when comparing DELs to libraries assembled by OBOC synthesis is the accessible chemical space. DELs need to be built utilizing DNA compatible chemical transformations, while OBOC libraries, in theory, have higher tolerability toward “harsh” reaction conditions. Roessler et al. tested and summarized some transformation that work on solid phase particles, but are incompatible with DNA.^71^ In practice, however, the DEL community has established an impressive number of DNA compatible chemical transformations, leading to a broad accessible chemical space.^13^ The AS–MS/MS workflows, reported so far, did not take advantage of the higher chemical tolerance of their OBOC synthesis. It has yet to be shown if this advantage can be leveraged to access libraries with a more diverse chemical space compared to DELs.

Most of the AS–MS/MS applications so far have focused on the identification of linear peptides. Cyclic peptides have a number of advantages over their linear counterparts:^12,86,87^ with their semirigid, preorganized structures, they often achieve higher binding affinity and selectivity. Also, they are more stable toward proteolytic digestion and can have enhanced cell penetration. For molecular biology based display technologies, the macrocyclization represents an engineering challenge, but the decoding of the genetic material remained unaltered. The challenge in AS–MS/MS, on the other side, is at the level of the mass spectrometry decoding step, which requires a linear sequence, for fragmentation. Individual examples show the possibility of solving this issue by using cleavable linkers.^5,81^ We predict that developments toward AS–MS/MS workflows with cyclic or bicyclic SELs will be pursued.

Peptides and peptidomimetics are seen as promising chemical modalities for targeting protein–protein interaction or challenging targets in general.^88^ On the other hand, small molecules usually have better pharmacokinetic properties and have better chances to become approved drugs. The AS–MS/MS platform has focused so far on peptidomimetic libraries. While the synthesis of combinatorial small molecules with drug-like properties would be possible, there is no available software for the automated decoding of selection hits resulting from such a library. As described above, automated peptide sequencing software has been a crucial technology for enabling advanced AS–MS/MS. A first important step toward drug-like molecule discovery has been the peptide encoded library (PEL) technology.^71^ However, the largest PEL reported by Roessler et al. contained only ∼40 thousand members. Any development enabling AS with ultralarge self-encoded small molecule libraries would be revolutionary.

Overall, the AS–MS/MS technology has a high potential to become a widespread early drug discovery technology applied in academic and industrial settings. The library synthesis and affinity selection basically require no specialized equipment. For the decoding, an advanced mass spectrometer is crucial. However, many universities have proteomics facilities that could be used for this purpose, obviating the need to purchase an additional instrument. With this, we predict that more groups will adopt the AS–MS/MS technology as a practical way to identify high affinity binders for disease related biomolecules or discover otherwise functional compounds.
